# Association Between Digital Biomarkers of Health and Anxiety: Systematic Review and Meta-Analysis

**DOI:** 10.2196/73812

**Published:** 2026-03-09

**Authors:** Yolanda Lau, Natalia Chemas, Heema Ajeet Gokani, Rachel Morrell, Harisd Phannarus, Claudia Cooper, Zuzana Walker, Harriet Demnitz-King, Natalie L Marchant

**Affiliations:** 1 Division of Psychiatry University College London London United Kingdom; 2 Centre for Preventive Neurology Wolfson Institute of Population Health Queen Mary University of London London United Kingdom; 3 Department of Preventive and Social Medicine Faculty of Medicine Siriraj Hospital Mahidol University Bangkok Thailand; 4 Centre for Psychiatry and Mental Health Wolfson Institute of Population Health Queen Mary University of London London United Kingdom; 5 Essex Partnership University NHS Foundation Trust Essex United Kingdom

**Keywords:** anxiety, digital, heart rate, machine learning, meta-analysis, physical activity, sleep, systematic review, wearable

## Abstract

**Background:**

Digital biomarkers are gaining interest as proxy markers for mental health, as they enable passive and continuous data collection. However, the association between digital biomarkers of health and anxiety, both generalized anxiety disorder and anxiety symptoms, remains unknown.

**Objective:**

This systematic review and meta-analysis examined the association between digital biomarkers of health obtained from wrist-worn wearables and anxiety in adults.

**Methods:**

Systematic literature searches were conducted across 6 databases, including unpublished gray literature. The final search was done on September 21, 2025. Cross-sectional or longitudinal studies investigating the association between digital biomarkers from wrist-worn wearables and anxiety were eligible. Studies using inferential statistics or machine learning methods were both eligible. Studies were excluded if participants received diagnoses of neurodegenerative disorders or physical health conditions. Two risk-of-bias tools were used: the National Heart, Lung, and Blood Institute assessment tool for inferential statistical studies, and the modified version of the Quality Assessment of Diagnostic Accuracy Studies-2 for machine learning studies. Whenever possible, effect sizes were combined across studies, for each digital biomarker of health separately, using random-effects meta-analyses. Sensitivity analyses were performed to assess whether results differed according to anxiety type (state or trait) and age group. Otherwise, studies were synthesized narratively.

**Results:**

A total of 44 studies from 42 articles were eligible. Among these, 36 studies used inferential statistical approaches for analysis (21 reporting sleep characteristics, 8 reporting physical activity, 2 reporting heart rate variability, and 5 reporting more than 1 type), and 8 studies used machine learning approaches. Sample size ranged from 17 to 170,320. Meta-analyses on 4 sleep metrics found no associations: sleep efficiency (Fisher *z*=–0.07, 95% CI –0.14 to 0.002; *P*=.06; PI –0.19 to 0.05), wake after sleep onset (Fisher *z*=0.13, 95% CI –0.04 to 0.30; *P*=.11; PI –0.15 to 0.41), total sleep time (Fisher *z*=0.009, 95% CI –0.01 to 0.03; *P*=.28; PI –0.02 to 0.03), and sleep onset latency (Fisher *z*=0.04, 95% CI –0.07 to 0.15; *P*=.08; PI –0.19 to 0.27). Qualitative syntheses revealed that lower physical activity levels and higher heart rate were associated with greater anxiety symptoms. Machine learning studies using wrist-worn wearable data alone showed varied performance, with predictive performance improving when wearable data were combined with other data sources.

**Conclusions:**

This is the first review to synthesize evidence from inferential statistical (mostly fair quality) and machine learning studies examining association between wearable-derived digital biomarkers and anxiety. Meta-analyses found no associations between sleep metrics and anxiety. Although based on limited studies, lower physical activity levels and elevated heart rate were associated with greater anxiety symptoms. Digital biomarkers may be more useful when integrated with other data sources (eg, self-report and clinical data) rather than used as stand-alone screening tools.

**Trial Registration:**

PROSPERO CRD42023409995; https://www.crd.york.ac.uk/PROSPERO/view/CRD42023409995

## Introduction

Anxiety is the most prevalent mental health condition globally, impacting 301 million people in 2019 [1]. Symptoms can include worry, restlessness, fatigue, difficulty concentrating, and sleep disturbances [2]. Among older adults, anxiety disorders and anxiety symptoms are common, with 1.2% to 28% experiencing anxiety disorders (generalized anxiety disorder [GAD] being the most common type) and 15% to 56% experiencing anxiety symptoms in community and clinical populations [3]. In older adults, both GAD and anxiety symptoms have been associated with increased risk of dementia [4-8]. Given the increasing global prevalence of dementia [9] and the lack of effective disease-modifying treatments, it is crucial to prioritize the identification and treatment of modifiable risk factors associated with dementia to reduce dementia cases. Therefore, early identification of anxiety in both the general population and older adults could have significant clinical implications.

Predominant methods to assess anxiety include the use of self-report questionnaires, clinical diagnoses, or a combination of both. These methods have several limitations. Self-report questionnaires measuring trait anxiety are retrospective and rely on an individual’s ability, insight, and willingness to respond accurately. Evidence suggests that self-report questionnaires can also be affected by recall bias and social desirability, potentially leading to misreporting of anxiety symptoms [10]. Clinical diagnoses require clinical expertise and are, therefore, time-consuming and resource-heavy. In addition, in older adults, anxiety is often underdiagnosed due to overlapping symptoms with physical health conditions [11]. Both methods only provide a snapshot of an individual’s symptomology unless assessed repeatedly, which can increase demands on both individuals and clinicians.

Leveraging digital biomarkers, defined as objective, quantifiable, physiological, and behavioral measures collected using digital devices [12], as proxy markers of anxiety has the potential to overcome the limitations outlined above. Digital biomarkers collect continuous objective data on various aspects of behavior and physiology.

While digital biomarkers can be measured using different technologies (eg, contactless sensors or smartphones), this review focuses on wrist-worn wearable devices, which are widely used by the general public and in existing research [13,14]. Wrist-worn wearables can measure physical activity (eg, different physical intensities), sleep (eg, sleep efficiency [SE]), and heart rate (eg, heart rate variability [HRV]). These variables have been linked to anxiety; for example, lower physical activity levels and more sleep disturbances have been associated with more anxiety symptoms and GAD [15,16]. There is also emerging evidence supporting the effectiveness of wearable-derived metrics in detecting mental health symptoms [17,18]. Studies also found that digital biomarkers collected from other devices can detect mental health symptoms. For instance, a study found that metrics from smartphones (including heart rate metrics) were able to predict changes in anxiety symptoms [19]. Similarly, studies using the Oura Ring also reported associations between sleep metrics and anxiety [20].

Two recent meta-analyses of machine learning studies examined the association between metrics collected from wearable devices and depression and anxiety, both of which are modifiable psychosocial risk factors of dementia [17,18]. They found that the models accurately classified individuals with and without depression in 89% of cases [17] and correctly identified individuals with and without anxiety in 82% of cases [18]. Similar wearable-derived metrics were used as predictors in anxiety and depression models (eg, activity, sleep, and heart rate). These meta-analyses only focused on machine learning studies; existing research also used inferential statistical methods.

Inferential statistical methods and machine learning models offer distinct advantages. Inferential statistical models provide interpretable results, allowing for hypothesis testing and quantifying associations between digital biomarkers of health and anxiety. In contrast, machine learning models often prioritize predictive accuracy and excel at capturing complex, nonlinear relationships that may not be easily captured through inferential statistical models. Together, these 2 approaches offer complementary insights.

Given the growing interest in using wearable-derived metrics in mental health and dementia research, this review aims to contribute to these research fields by systematically reviewing existing studies that have investigated the association between metrics collected from wrist-worn wearables and anxiety using inferential and machine learning statistical approaches.

## Methods

### Design

This review was conducted in line with the PRISMA (Preferred Reporting Items for Systematic Reviews and Meta-Analyses) recommendation [21] and registered with PROSPERO (International Prospective Register of Systematic Reviews) in March 2023 (CRD42023409995). The PRISMA 2020 checklist [22] (Multimedia Appendix 1), the PRISMA Abstract checklist [22] (Multimedia Appendix 2), and the PRISMA-S (Preferred Reporting Items for Systematic Reviews and Meta-Analyses–Search Extension) checklist [23] (Multimedia Appendix 3) were used.

### Search Strategy

A total of 5 databases (Medline, Embase, PsycINFO, Web of Science, and CINAHL) were systematically searched. Further, unpublished gray literature searches were conducted with ProQuest Dissertations and Theses Global. Following published recommendations [24], we also checked the first 300 papers on Google Scholar to supplement the gray literature search. Finally, to supplement the electronic search, references of eligible papers were reviewed to identify any additional eligible studies.

The search strategy combined 2 search term strings with “AND.” Each string reflected a concept related to the research question: (1) digital biomarkers and (2) psychosocial risk factors of dementia (Multimedia Appendix 4). The search strategy was informed by previous reviews in related research areas [17], but all search terms and strategies were developed specifically for this review and adapted for each database. There were no restrictions imposed on the date of publication. This review did not restrict the database searches to human studies and studies published in English, and no filters were used. The initial searches were conducted on April 27, 2023; a subsequent search was done on September 24, 2024, and a final search focusing only on anxiety was done on September 21, 2025. While the PROSPERO registration included multiple psychosocial risk factors, this systematic review focused specifically on anxiety, due to the large number of articles that made it past full-text screening. As all eligible anxiety studies in earlier searches were identified via anxiety-specific terms, a final search restricted to anxiety-related search terms was conducted in 2025.

### Study Selection

The web platform Covidence (Veritas Health Innovation, 2020) was used for deduplication and to coordinate multiuser title-abstract and full-text screening. Two reviewers independently screened titles and abstracts of all the identified studies (YL screened 100% of the articles, and NC, HAG, RM, and HD-K collectively screened 100%), followed by full-text screening. Any disagreements were resolved by a third reviewer (NM or HD-K), with HD-K only resolving conflicts for articles she had not screened at the title-abstract screening stage. Interrater agreement was assessed using the Cohen κ coefficient.

In accordance with the PROSPERO registration, our search strategies encompassed a range of psychosocial risk factors for dementia. However, given the large number of eligible articles that made it through to the full-text screening stage, we decided to categorize these articles into different psychosocial risk factors before the data extraction stage.

This systematic review focused on anxiety, both clinical anxiety and anxiety symptoms. The PECOS (Population, Exposure, Control, Outcome, and Study design) framework was used to structure the study selection. Studies were selected if they (1) involved adults with a mean age of >18 years (Population); (2) reported data from research- or consumer-grade wrist-worn devices (Exposure); (3) did not require a control group, although studies including participants without anxiety were considered as comparators when applicable (Control); (4) assessed GAD or anxiety symptoms using standardized assessments (eg, self-report questionnaires or clinical diagnoses [Outcome]); and (5) used cross-sectional or longitudinal designs (Study design). Studies published in an English-language, peer-reviewed journal or thesis/dissertation, and studies that examined the relationship between anxiety and digital biomarkers via inferential statistics or machine learning methods were also eligible. Studies were excluded if they (1) focused on a sample that received diagnoses of neurodegenerative disorders or physical health conditions (however, studies were eligible if participants without these conditions were analyzed separately), and (2) primarily focused on participants with a psychiatric comorbidity, neurological disorder, or stroke (>50% of the sample).

### Data Extraction

A standardized form was created to extract the following data from eligible inferential statistical studies: (1) authors and year of publication; (2) study characteristics, including type of study (ie, cross-sectional or longitudinal); (3) demographic information; (4) characteristics of digital biomarkers, including digital biomarker type and devices used for data collection; (5) anxiety measurement, and (6) data required for meta-analysis (eg, mean difference and correlation coefficients). For studies using machine learning approaches, another standardized form was created to extract the following details: (1) authors and year of publication, (2) demographic information, (3) ground truth assessment, (4) characteristics of digital biomarkers (5) machine learning task, (6) predictor features, (7) algorithms, (8) validation method, and (9) model performance.

Where necessary, authors were contacted to request additional information.

### Quality Assessments

#### Inferential Statistical Studies

The National Heart, Lung, and Blood Institute assessment tool was used [25]. This quality assessment tool comprises 14 criteria, which are used to evaluate the validity and reliability of studies. For cross-sectional studies, 4 criteria that are relevant to longitudinal study designs are not applicable and were therefore omitted [26]. Scores were averaged, and each study was categorized as having a “Good” (≥80), “Fair” (50%-80%), or “Poor” (50%) rating, as specified in the guidelines.

#### Machine Learning Studies

Currently, there is no validated quality assessment tool for machine learning studies. We used a modified version of the validated Quality Assessment of Diagnostic Accuracy Studies 2 [27], which evaluates the risk of bias of studies across 4 domains (participants, index test [artificial intelligence algorithms], reference standard [ground truth], and analysis), and evaluates applicability concerns across 3 domains (excluding the analysis domain) [17].

Two independent reviewers assessed the quality of each eligible study. Any disagreements were resolved by a third reviewer.

### Synthesis and Analysis

Effect sizes were combined across studies, separately for each type of digital biomarker of health, using random-effects meta-analyses. When effect sizes were unavailable, other available data were used for calculations (eg, *t* test statistic). Random-effects meta-analyses were used as they take into account between-study heterogeneity [28]. For meta-analysis based on a limited number of studies (<20 studies [29]), we applied the Hartung-Knapp-Sidik-Jonkman adjustments when calculating CIs and *P* values, which are recommended for small meta-analyses [30]. Prediction intervals for random-effects meta-analyses were also reported [31].

To ensure only 1 effect size from each included study was used in the primary meta-analysis, studies were selected based on an a priori determined hierarchy. Specifically, we prioritized (1) the largest sample size, (2) unadjusted over adjusted estimates, and (3) trait (chronic/ongoing) over state (present moment) anxiety, when more than 1 study used the same cohort. Trait anxiety refers to the general tendency to respond with anxiety across various situations, whereas state anxiety is a temporary response reflecting the present moment [32]. Since trait anxiety reflects a chronic condition with long-term implications and offers a more stable measure of general anxiety levels, it was the focus of our analysis.

Sensitivity analyses were conducted to examine whether results differed according to anxiety type (trait and state anxiety) and age group (young, middle, and older).

For each meta-analysis, heterogeneity was assessed using τ^2^ (between-study variance) and *I*^2^ (proportion of total variation in study estimates that is due to heterogeneity [33]). When there were 10 or more studies included in a meta-analysis, funnel plots would be used to assess small-study effects [34].

All analyses were conducted using the *metafor* package in R software (version 4.2.2; R Foundation for Statistical Computing).

Qualitative synthesis was conducted for studies using inferential statistical approaches that were not included in the meta-analysis. These studies were synthesized based on the type of digital biomarkers of health examined (ie, sleep metrics and activity metrics). On the other hand, studies using machine learning approaches were not meta-analyzed and were synthesized qualitatively, ordered by performance metrics (ie, accuracy, sensitivity, precision, and *F*_1_-score).

## Results

### Study Selection

**Figure 1:**
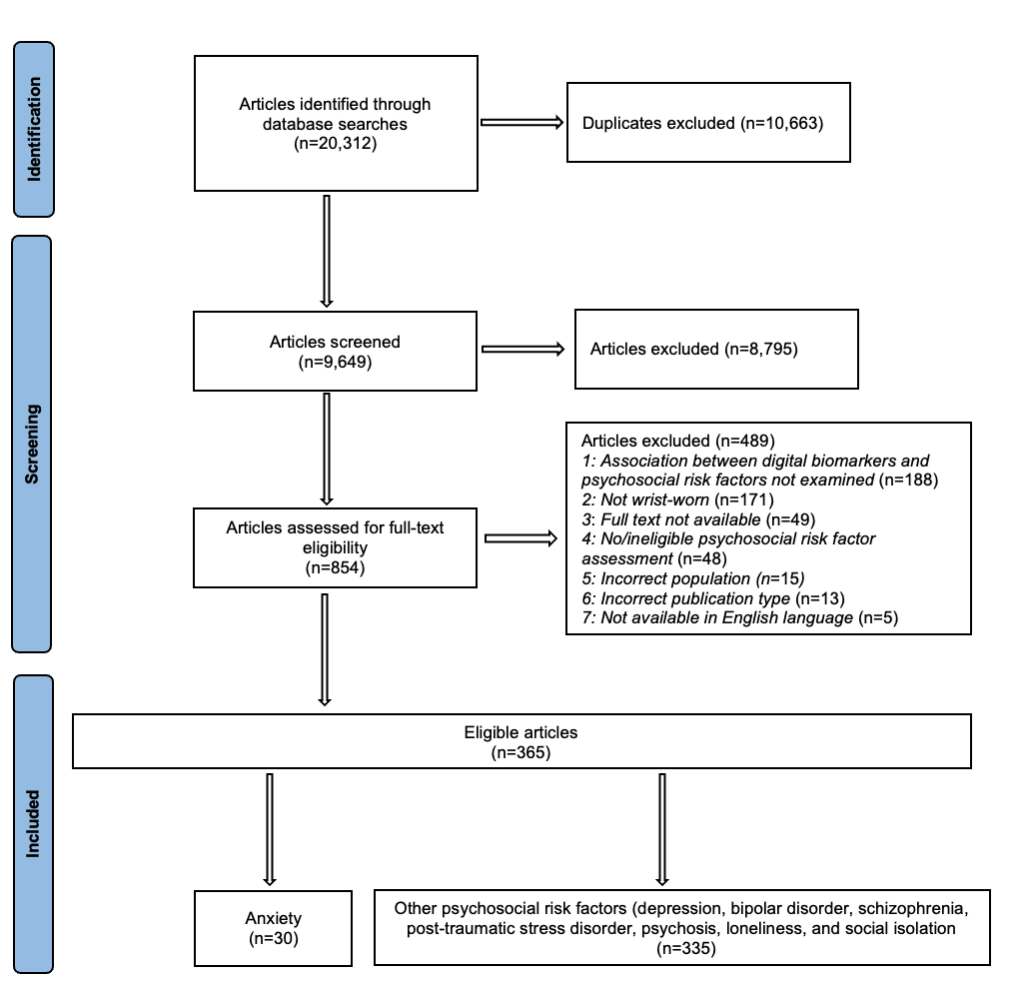
PRISMA (Preferred Reporting Items for Systematic Reviews and Meta-Analyses) flow diagram illustrating the systematic process.

After the removal of duplicates, title/abstract screening, and full-text review, 365 articles were eligible (Figure 1). Of these, 47 articles focused on anxiety. Interrater reliability was high, with Cohen κ of 0.73 (substantial agreement) and 0.83 (almost perfect agreement) at the title/abstract screening and full-text review stages, respectively.

### Study and Participant Characteristics

#### Overview

When an article reported outcomes from different cohorts or separately analyzed different populations (eg, male and female), each result was treated as a different study derived from the same article. This resulted in 44 studies from 42 articles. Of these, 36 (81.8%) studies used inferential statistical approaches for analysis, while 8 (18.2%) studies used machine learning approaches.

#### Studies Using Inferential Statistical Approaches

Characteristics of studies that used inferential statistical approaches are presented in Table 1. There was a total of 36 studies from 35 articles (cross-sectional: k=21, 58.3%; longitudinal: k=11, 30.6%; both cross-sectional and longitudinal: k=4, 11.1%). The study sizes ranged from 14 to 170,320 participants (median 189, IQR 79-2276). Most of the studies assessed trait anxiety or clinically diagnosed anxiety (k=19, 52.8%), 10 (27.8%) assessed state anxiety, and 7 (19.4%) assessed both state and trait anxiety. The majority of studies were conducted in young adults (k=18, 50%), 8 (22.2%) in middle-aged adults, 8 (22.2%) in older adults, and 2 (5.6%) did not report mean age data.

**Table 1:** Characteristics of samples eligible for systematic review and/or meta-analysis of studies using inferential statistical approaches. Most studies were cross-sectional (k=21), with sample sizes ranging from 14 to 170,320 (median 189, IQR 79-2276); most assessed trait or clinically diagnosed anxiety (k=21) and were conducted in young adults (k=18).

Study (location)	Study design (follow-up time)	Participants, N	Population type	Age (years), mean (SD) Sex (female), % Education, mean (SD) or categories % Ethnicity (White), %	Anxiety measure (type)	Wearable device (research/consumer-grade)	Digital biomarker type (subtype)
Ahn et al, 2024 (United States) [35]	Cross-sectional	33	Family caregivers	Age: 61.27 (11.2) Female: 84.8% Education: NR^a^ White: 90.9%	GAD-7^b^ (trait)	ActiGraph GT9X Link (research)	Sleep (SE^c^)
Bullis, 2016 (United States) [36]^d^	Cross-sectional	30	Clinical samples and healthy controls	Age: 28 (9.0) Female: 70% Education: high school graduate 6.7%; partial college 16.7%; college graduate 46.7%; graduate 30% White: 73.3%	Clinical diagnosis (*DSM-5*^e^—trait)	Actiwatch (research)	Sleep (SOL^f^, TST^g^, SE, and WASO^h^)
Bullis, 2016 (United States) [36]^c^	Longitudinal (14 days)	30	Clinical samples and healthy controls	Age: 28 (9.0) Female: 70% Education: high school graduate 6.7%; partial college 16.7%; college graduate 46.7%; graduate 30% White: 73.3%	Clinical diagnosis (*DSM-5*—trait) and daily symptom ratings (Ecological Momentary Assessment—state)	Actiwatch (research)	Sleep (SOL, TST, SE, and WASO)
Casanova et al, 2023 (United Kingdom) [37]	Cross-sectional	95,776	General population	Age: NR Female: NR Education: NR White: NR	GAD-7 (trait)	Axivity AX3 (research)	Activity (physical activity and SB^i^)
Cox et al, 2018 (United States) [38]	Cross-sectional	138	Undergraduate students and community adults	Age: 22.5 (9.2) Female: 75.5% Education: NR White: 57.6%	Momentary anxiety (state)	ActiGraph wGT3X-BT (research)	Sleep (TST)
Cox et al, 2018 (United States) [38]	Longitudinal (9 days)	138	Undergraduate students and community adults	Age: 22.5 (9.2) Female: 75.5% Education: NR White: 57.6%	Momentary anxiety (state)	ActiGraph wGT3X-BT (research)	Sleep (TST)
D’Aurizio et al, 2023 (Italy) [39]^c^	Cross-sectional	60	Prisoner and healthy control	Age: 36 (8.46) Female: 0% Education: 5-8 years White: NR	STAI^j^ (trait and state)	Actiwatch Spectrum Plus (research)	Sleep (SOL, SE, TST, and WASO)
Dillon et al, 2018 (Ireland) [40]	Cross-sectional	397	Patients (population-representative random sample, from a primary care center)	Age: 59.6 (5.5) Female: 53.9% Education: primary 26.7%, secondary/tertiary 73.3% White: NR	HADS-A^k^ (state)	GENEActiv accelerometer (research)	Activity (SB, LPA^l^, and MVPA^m^)
Doane et al, 2015 (United States) [41]^c^	Cross-sectional	82	Adolescent transition to college	Age: 18.1 (0.4) Female: 76% Education: NR White: 54%	DASS-A^n^ (state)	Actiwatch Score (research)	Sleep (sleep start time variability, SOL, SE, and TST)
Doane et al, 2015 (United States) [41]^c^	Longitudinal (1 year)	Follow-up 1: 76 Follow-up 2: 71	Adolescent transition to college	NR	DASS-A (state)	Actiwatch Score (research)	Sleep (sleep start time variability, SOL, SE, and TST)
El-Sheikh et al, 2013 (United States; female) [42]^c^	Cross-sectional	135	Cohabiting couples	Age: 36.5 (5.9) Female: 100% Education: NR White: NR	BAI^o^ (trait)	Octagonal Basic Motionloggers (research)	Sleep (TST, SE, and SOL)
El-Sheikh et al, 2013 (United States; male) [42]^c^	Cross-sectional	135	Cohabiting couples	Age: 39.4 (7.3) Female: 0% Education: NR White: NR	BAI (trait)	Octagonal Basic Motionloggers (research)	Sleep (TST, SE, and SOL)
Facer-Childs et al, 2022 (Australia) [43]	Cross-sectional	68	Elite football league athletes	Age: 23.3 (3.4) Female: 0% Education: NR White: NR	GAD-7 (trait) and DASS-A (state)	GENEActiv actigraphy devices (research)	Sleep (snooze time, SE, TST, and TIB^p^)
Feng et al, 2021 (United States) [44]	Cross-sectional	14	Ophthalmology residents	Age: 30.2 (2.5) Female: 57.1% Education: NR White: NR	DASS-A (state)	Fitbit Alta HR (consumer)	Sleep (lower average sleep on call) and activity (average daily exercise)
Fuller-Rowell et al, 2024 (United States) [45]^c^	Cross-sectional	874	Small towns and semirural communities	Age: 41.0 (7.0) Female: 67% Education: NR White: 67%	BAI (trait)	Octagonal Basic Motionloggers (research)	Sleep (TST, night-to-night variability, SE, and SOL)
Hofman et al, 2022 (Netherlands) [46]	Cross-sectional	1943	General population	Age: 70.9 (9.3) Female: 51.6% Education: primary 6%, lower 36.7%, further/intermediate 30.6%, higher 25.4% White: NR	HADS-A (state)	GENEActiv (research)	Sleep (TST) and activity (SB, LPA, and MVPA)
Huang et al, 2022 (China) [47]	Cross-sectional	321	College students	Age: 19.7 (1.2) Female: 55.8% Education: NR White: NR	SAS^q^ (state)	Axivity AX3 (research)	Activity (SB-total, weekend, and weekdays)
Lee et al, 2021 (United States, Australia, South Korea) [48]^c^	Cross-sectional	17	Elite esports athletes	Age: 20 (3.5) Female: 0% Education: NR White: NR	STAI (state)	Readiband V5 (research)	Sleep (TST, SOL, WASO, SOT^r^, and WT^s^)
Montanari et al, 2025 (France) [49]	Cross-sectional	211	Older adults	Age: 60-70 years: 105 (50%); 71-80 years: 89 (42%); >80 years: 17 (8.1%) Female: 36% Education: lower education 31%, secondary education 32%, higher education (37%) White: NR	STAI (trait and state)	Actigraph GT3X+ (research)	Sleep (TST, WASO, and SE)
Nguyen et al, 2023 (Australia) [50]^c^	Cross-sectional	101	Newly recruited paramedics	Age: 26.0 (5.5) Female: 52% Education: NR White: NR	GAD-7 (trait)	Actiwatch Spectrum or Spectrum PRO (research)	Sleep (TST, WASO, SOL, and SE)
Nguyen et al, 2023 (Australia) [50]^c^	Longitudinal (6 months)	101	Newly recruited paramedics	Age: 26.0 (5.5) Female: 52% Education: NR White: NR	GAD-7 (trait)	Actiwatch Spectrum or Spectrum PRO (research)	Sleep (TST, WASO, SOL, and SE)
Spira et al, 2008 (United States) [51]^c^	Cross-sectional	59	Adults with primary insomnia	Age: 63.8 (6.9) Female: 74.6% Education: mean 16.3 (SD 2.3) years White: 78%	STAI (trait and state)	Actiwatch-L (research)	Sleep (WASO, SE, and SOL)
Spira et al, 2009 (United States) [52]^c^	Cross-sectional	3040	Community-dwelling older women	Age: 83.6 (3.8) Female: 100% Education: mean 12.9 (SD 2.6) White: 89.3%	Goldberg Anxiety Scale (trait)	SleepWatch-O (research)	Sleep (TST, SE, time awake after sleep onset, SOL, and napping time)
Stremler et al, 2017 (Canada) [53]	Cross-sectional	118	Parents of critically ill hospitalized children	Age: 34.1 (7.2) Female: 62.7% Education: <high school 5.1%, high school 22%, university/college 53.4%, postgraduate 17.8%, unknown 1.7% White: 69.4%	STAI (state)	Octagonal Basic Motionlogger actigraphs (research)	Sleep (TST, number of nocturnal awakenings, and individual sleep variability)
Swanson et al, 2023 (United States) [54]^c^	Cross-sectional	1197	Postmenopausal women	Age: 65.5 (2.6) Female: 100% Education: less than high school 5.2%, high school 15.3%, some college 30.7%, college 22.6%, post-college 26.2% White: 45.7%	GAD-7 (trait)	Actiwatch-2 (research)	Sleep (sleep irregularity and sleep midpoint outside 2-4 AM)
Uchida and Kurosawa, 2025 (Japan) [55]	Cross-sectional	50	University students	Age: 20.58 (1.34) Female: 64% Education: NR White: NR	Japanese version of STAI (trait and state)	Fitbit Charge 5 (consumer)	Sleep (TST, bedtime, and wake time)
Windmill et al, 2024 [56]	Cross-sectional	83	General population	Age: 24 (5.1) Female: 65.7% Education: NR White: NR	PANAS^t^ (state and trait), STAI (state and trait), BAI (state)	GENEActiv, ActivInsights (research)	Sleep (SE and TST)
Walters et al, 2020 (Australia) [57]	Cross-sectional	110	Without sleep disorders	Age: 32.3 (12.4) Female: 50.9% Education: NR White: NR	BAI (trait)	Respironics Actiwatch Spectrum Pro (research)	Sleep (SE)
Zhang et al, 2025 (United States) [58]	Cross-sectional	10,129	General population	Age: median 48.0 (IQR 36.0-58.0) Female: 69.3% Education: NR White: 91.4%	GAD-7 (trait)	Fitbit (consumer)	Sleep (eg, TST, wake time, sleep start, percentage of long sleep days, and percentage of short sleep days) Physical activity (eg, steps, sedentary duration, light activity duration, and total active duration) Heart rate metrics (eg, average heart rate and SD)
Zheng et al, 2024 (United States) [59]	Longitudinal (4.5 years)	6785	General population	Age: median 50.2 (IQR 35.7-61.5) Female: 70.8% Education: NR White: 83.6%	Electronic health records	Fitbit (consumer)	Sleep (TST, sleep stages, and sleep regularity)
Bai et al, 2024 (United States) [60]	Longitudinal (16 weeks)	167	University students	Age: NR Female: 79% Education: NR White: 92.2%	Daily survey measuring anxiety	Apple Watch (consumer)	Physical activity (MVPA and steps)
Jo et al, 2024 (Korea) [61]	Longitudinal (4 weeks)	47	Young adults	Age: 28.7 (5.9) Female: 48.9% Education: NR White: NR	GAD-7 (trait)	Samsung Galaxy Active 2 (consumer)	HRV^u^ (time-domain measures: RMSSD^v^, SDNN^w^, SDSD^x^, and PNN50^y^; frequency-domain measures: LF^z^, HF^aa^, and LF/HF^ab^)
Kandola et al, 2021 (United Kingdom) [62]	Longitudinal (2 years)	60,235	General population	Age: 55.9 (7.7) Female: 56% Education: college/university degree 48%; a-level/as levels/equivalent 14%; GCSEs^ac^/O-levels/equivalent 19%; CSEs^ad^/equivalent 3.4%; NVQ^ae^/HND^af^/equivalent 4.9%; other professional qualifications 4.9%; none (6.1%) White: 98%	GAD-7 (trait)	Axivity AX3 (research)	Activity (SB, LPA, and MVPA)
Liu et al, 2025 (China) [63]	Longitudinal (7-9 years)	84,570	General population	Age: 56.2 (7.8) Female: 55.5% Education: college/university degree 34.8%, other levels of education (64%), unknown (1.2%) White: 96.8%	Patient Health Questionnaire, *ICD-10*^ag^ codes (trait)	Axivity AX3 (research)	Physical activity (MVPA)
McDuff et al, 2025 (United States) [64]	Longitudinal (4 weeks)	237	General population	Age: 45.1 (11.8) Female: 67.5% Education: NR White: 87.7%	GAD-7 (trait)	Fitbit Sense 2TM (consumer)	Skin conductance level, heart rate, skin temperature, and step count
Okawara et al, 2024 (Japan) [65]	Longitudinal (60 days)	279	Office workers	Age: 42.0 (10.0) Female: 16.1% Education: NR White: NR	STAI trait subscale (trait)	Apple Watch (consumer)	HRV (HF, LF, LF/HF ratio, and LnccL/H^ah^)
Presby et al, 2025 (Switzerland, United States, Australia) [66]	Longitudinal (13 months)	170,320	General population	Age: 37.39 (10.36) Female: 33% Education: NR White: 33.4%	Generalized Anxiety Disorder 2-item (trait)	WHOOP strap version 3.0 and 4.0 (consumer)	Sleep (TST, SE, SD of TST, sleep consistency, sleep start, and wake start) Heart rate (HRV and resting heart rate) Physical activity (total activity)
Straus et al, 2023 (United States) [67]	Longitudinal (8 weeks)	2021	Trauma survivors from the emergency department	Age: 35.8 (13.0) Female: 62.2% Education: <high school 12%, high school graduate 25.6%, some college 41.3%, college graduate 20.7% White: 34.3%	Smartphone-based questionnaires (state—measured 6 times over the 8-week period)	Verily Life Sciences (research)	Sleep (number of transitions between sleep and wake)
Wang et al, 2025 (China) [68]	Longitudinal (12.6 years)	91,800	General population	Age: 61.8 (7.9) Female: 56.1% Education: college above 43.7%, college below 56.3% White: 97%	*ICD-10* (trait)	Axivity AX3 (research)	Physical activity (LPA, moderate intensity, vigorous intensity, and MVPA)
Yu et al, 2025 (Japan) [69]	Longitudinal (2 years)	71,556	Healthy adults aged 40-69 years	Age: 62.11 (7.83) Female: 54.4% Education: college or higher 44.4% White: 97.3%	GAD-7 (trait)	Axivity AX3 (research)	Physical activity (MVPA, LPA, and SB)

^a^NR: not reported.

^b^GAD-7: Generalized Anxiety Disorder-7.

^c^SE: sleep efficiency.

^d^Studies included in meta-analyses.

eDSM-5: Diagnostic and Statistical Manual of Mental Disorders (Fifth Edition).

^f^SOL: sleep onset latency.

^g^TST: total sleep time.

^h^WASO: wake after sleep onset.

^i^SB: sedentary behavior.

^j^STAI: State-Trait Anxiety Inventory.

^k^HADS-A: Hospital Anxiety and Depression Scale-Anxiety section.

^l^LPA: light-intensity physical activity.

^m^MVPA: moderate to vigorous physical activity.

^n^DASS-A: Depression, Anxiety, and Stress Scale-Anxiety Subscale.

^o^BAI: Beck Anxiety Inventory.

^p^TIB: time in bed.

^q^SAS: Self-Rating Anxiety Scale.

^r^SOT: sleep onset time.

^s^WT: wake time.

^t^PANAS: Positive and Negative Affect Schedule.

^u^HRV: heart rate variability.

^v^RMSSD: root mean square of successive differences between RR intervals (intervals between successive R peaks) interval differences.

^w^SDNN: SD of NN intervals (intervals between successive normal heartbeats).

^x^SDSD: SD of RR interval intervals.

^y^PNN50: percentage of successive RR intervals that differ by more than 50 ms.

^z^LF: absolute power of the low-frequency band.

^aa^HF: absolute power of the high-frequency band.

^ab^LF/HF: ratio of LF to HF power.

^ac^GCSE: General Certificate of Secondary Education.

^ad^CSES: Certificate of Secondary Education.

^ae^NVQ: National Vocational Qualification.

^af^HND: Higher National Diploma.

agICD-10: International Statistical Classification of Diseases, Tenth Revision.

^ah^LnccL/H: log-transformed coefficient of component variance of the low-frequency component to high-frequency component power ratio.

Most studies assessed only sleep (k=21, 58.3%), 8 (22.2%) studies assessed only physical activity, 2 (5.6%) studies assessed only HRV, and 5 (13.9%) studies assessed more than one type. The majority of sleep studies assessed the following 4 sleep metrics: total sleep time (TST; ie, total time spent in bed), wake after sleep onset (WASO; ie, time spent awake after initially falling asleep), SE (ie, ratio of TST to the total time spent in bed), and sleep onset latency (SOL; ie, time it takes to fall asleep after going to bed). Regarding physical activity, sedentary behavior, light physical activity, and moderate to vigorous physical activity (MVPA) were most commonly assessed.

#### Studies Using Machine Learning Approaches

Characteristics of studies using machine learning approaches are presented in Table 2. A total of 8 studies from 7 articles were included. The study sizes ranged from 40 to 727 participants (median 326, IQR 66-410). All studies used algorithms trained with a labeled dataset to select predictors for predicting anxiety (ie, supervised learning). Included studies used a variety of digital biomarkers of health as predictors in their models, for example, HRV, sleep, and activity.

**Table 2:** Characteristics of samples for studies using machine learning approaches. All studies used supervised learning, with sample sizes ranging from 40 to 727 (median 326, IQR 66-410).

Study	Population type	Participants, N	Age (years), mean (SD) Sex (female), %	Ground truth assessment, anxiety measure (type)	Wearable device (consumer or research-grade)	Machine learning task	Predictors	Algorithms	Validation method
Coutts et al, 2020 (United Kingdom) Trial 1 [70]	University students	68	Age: 21 (NR^a^) Female: 64%	DASS-A^b^ (state) and STAI^c^ (trait and state)	Biobeam band (research)	Binary classification (supervised)	Heart rate variability	Deep neural networks (LSTM^d^)	Train-test split: training (80%), test (10%), validation (10%)
Coutts et al, 2020 (United Kingdom) Trial 2 [70]	University students	584	Age: NR Female: NR	DASS-A (state) and STAI (trait and state)	Biobeam band (research)	Binary classification (supervised)	Heart rate variability	Deep neural networks (LSTM)	Train-test split: training (80%), test (10%), validation (10%)
Fukuda et al, 2020 (Japan) [71]	Office workers	60	Age: NR Female: NR	DAMS^e^ (state)	Fitbit Charge 3 (Consumer)	Binary classification (supervised)	Sleep actigraphy data (13 features, eg, total sleep time and number of wake)	RF^f^	Leave-one-person-out cross-validation
He et al (2025) [72]	University students	40	Age: 21.7 (3.11) Female: 52.5%	STAI Y6 (state)	E4 Empatica Hexoskin smart shirt	Binary classification (unsupervised)	Wrist-worn wearable: skin temprature and EDA^g^ Smart shirt: ECG^h^	SVM^i^, RF, KNN^j^, naïve Bayes	Leave-one-participant-out
Lee et al, 2022 (Korea) [73]	Older adults with mild cognitive impairment	352	Age: 72.48 (5.9) Female: 73%	Clinical diagnosis (Trait)	Fitbit Alta HR2 (Consumer)	Binary classification (supervised)	Model 1: 24-hour activity rhythms and sleep pattern^k^ Model 2: Model 1+ minimal K-GAI^l^ (5 items)	Logistic regression, SVM, RF, GBM^m^	10-fold stratified cross-validation
Lee et al, 2024 (Korea) [74]	Older adults with mild cognitive impairment	352	Age: 72.5 (5.9) Female: 73%	Clinical diagnosis (Trait)	Fitbit Alta HR2 (Consumer)	Binary classification (supervised)	Model 1: activity, sleep Model 2: activity + minimal K-GAI	Convolutional neural network, LSTM, residual network	Cross-validation
Saylam et al, 2023 (United States) [75]	Office workers	727	Age: NR Female: NR	Single item^n^ (state)	Garmin smartwatch (consumer)	Regression	Activity, stress, sleep, heart rate	RF, XGBoost^o^, LSTM	80% training, 20% testing
Saylam et al, 2024 (United States) [76]	College students	Baseline: ~700 Follow-up: 300	Age: NR Female: NR	BAI^p^	Fitbit (consumer)	Regression	Activity and sleep	RF, XGBoost, LSTM	Training: 5 semesters Validation: 2 semesters Testing: 2 semesters

^a^NR: not reported.

^b^DASS-A: Depression, Anxiety, and Stress Scale-Anxiety Subscale.

^c^STAI: State Trait Anxiety Inventory

^d^LSTM: Long Short-Term Memory networks.

^e^DAMS: Depression and Anxiety Mood Scale.

^f^RF: random forest.

^g^EDA: electrodermal activity.

^h^ECG: electrocardiogram.

^i^SVM: Support Vector Machine.

^j^KNN: k-nearest neighbors.

^k^Model 1: 24-hour activity rhythms (interdaily stability [the stability of an activity rhythm of a daily pattern], intra-daily variability [the variability of the activity rhythms throughout the day], dominant rest phase onset [the start time of the 5-hour period with the least activity within 24 hours], and sleep patterns (total sleep time, sleep onset latency, wake after sleep onset, and sleep quality).

^l^K-GAI: Korean version of Geriatric Anxiety Inventory.

^m^GBM: gradient boosting machine.

^n^Single-item question “Please select the response that shows how anxious you feel at the moment. Response scale: 1 (not at all anxious), 2 (a little anxious), 3 (moderately anxious), 4 (very anxious), and 5 (extremely anxious).”

^o^XGBoost: extreme gradient boosting.

^p^BAI: Beck Anxiety Inventory.

### Quality Assessment

#### Studies Using Inferential Statistical Approaches

For studies that conducted both cross-sectional and longitudinal analyses, quality assessment was based on the longitudinal studies. Of the 36 studies, 12 (33.3%) received a “Good” quality rating; the remainder received a “Fair” rating (k=24, 66.7%; Multimedia Appendix 5).

#### Studies Using Machine Learning Approaches

The quality assessment tool for studies using machine learning approaches assesses the risk of bias across 4 domains: participants, index test, reference standard, and analysis. A total of 5 studies were rated as having a high risk of bias in the participants’ domain. For the index test, ground truth, and analysis, all studies were rated as either low risk or unclear. There were either unclear or low concerns regarding the applicability of the studies to our review question across the participants, index test, and reference standard domains (Multimedia Appendix 5 [70-76]).

### Qualitative Synthesis of Inferential Statistical Studies

#### Sleep

A total of 8 studies investigated the association between sleep metrics not included in the meta-analyses and anxiety. Sleep start variability [41], sleep onset time [48], wake time [48,55,58], bedtime [55,58], number of nocturnal awakenings [53], sleep variability [53], and sleep timing [54] were not associated with anxiety symptoms. Lower average sleep among doctors who were on call was negatively associated with anxiety symptoms [44]. Other studies found that greater sleep irregularity [54], greater sleep duration variability [45,58], higher sleep start variability [58], greater wake time variability [58], later wake time [58], and a higher percentage of long (>10 hours) and short (<6 hours) sleep days [58] were associated with more anxiety symptoms.

A total of 7 studies reported the longitudinal association between sleep and anxiety [36,38,41,50,59,66,67]. While 1 study found no association between baseline sleep metrics (ie, SE, WASO, SOL, and TST) and anxiety at 6 months follow-up in paramedics [50], the majority of studies did observe an association over varying periods of time. Lower TST compared to an individual’s personal TST average was associated with higher anxiety levels on the next day [38]. Anxious mood also predicted next day’s poorer sleep (ie, lower SE, longer WASO, and less TST) in individuals with clinical anxiety, but not in healthy controls [30]. Worsening sleep continuity (measured by the number of transitions between sleep and wake) was associated with worsening anxiety over an 8-week period [67], lower SE and longer SOL were associated with more anxiety symptoms at 1 year follow-up [41], and sleep patterns (increased sleep irregularity, decreased deep and rapid eye movement (REM) sleep percentage, and increased light sleep percentage) captured through continuous monitoring over a median period of 4.5 (IQR 2.5-6.5) years were associated with increased odds of GAD [59]. Additionally, they identified a nonlinear, J-shaped relationship between average daily sleep duration and GAD—compared with the median average daily sleep of 6.8 hours, individuals with an average daily sleep duration of 5 hours or 10 hours had increased odds of GAD. One study found that more consistent wake and sleep times were associated with lower anxiety symptoms [66].

#### Activity

A total of 6 studies investigated the association between sedentary behavior and anxiety [37,40,46,47,58,62]. While 1 study found no association between sedentary behavior and anxiety symptoms [47], the remainder did observe associations. Individuals with moderate to severe anxiety symptoms in primary care settings spent more time in sedentary activity per day than participants with no significant anxiety [40]. This finding was supported by a large study in the UK general population [58]. A study using isotemporal substitution models (which assess the effect of hypothetically replacing 1 activity with another while keeping the total time constant) found similar findings; they found that replacing MVPA with sedentary behavior was associated with higher odds of having clinically relevant anxiety symptoms [46]. Another study found that while sedentary time was not associated with current GAD and anxiety symptoms [37], it was associated with lower odds of lifetime GAD, and in males when stratified by sex [37]. The association between sedentary behavior and anxiety symptoms was also supported in a longitudinal study, with more baseline sedentary behavior associated with more anxiety symptoms at follow-up [62].

A total of 5 studies examined the association between light activity, MVPA, and anxiety [40,46,58,60,62] and reported mixed findings. Regarding light physical activity, 1 study found that lower light activity duration was associated with more anxiety symptoms. Consistent with this finding, 1 study found that individuals with moderate to severe anxiety symptoms spent less time in light physical activity than those without significant levels of anxiety [40]. Using isotemporal substitution models, 1 study found that replacing sedentary behavior with light physical activity was associated with a decrease in anxiety symptoms [40], while another study found no association [46], and a longitudinal study (UK Biobank) found that replacing sedentary behavior with light physical activity was associated with lower anxiety symptoms at follow-up [62]. Regarding MVPA, 1 study found that replacing sedentary behavior with MVPA was associated with lower odds of clinically relevant anxiety symptoms [46], but 1 study found no association [40]. One longitudinal study found that replacing baseline sedentary behavior with MVPA was associated with lower anxiety symptoms at follow-up [62], and another found that more anxiety symptoms (measured using a daily survey) were associated with lower MVPA [60].

A total of 3 studies examined associations between physical activity and anxiety using data from the UK Biobank [63,68,69]. One study reported an L-shaped association, where higher levels of moderate-intensity physical activity and vigorous-intensity physical activity were associated with lower incident anxiety [68]. Another study also identified a nonlinear relationship, with higher levels of MVPA and total physical activity associated with reduced anxiety risk, whereas light physical activity and sedentary behavior were not associated with anxiety [69]. The third study found that, compared with inactive individuals, those who were regularly active during the week or active only during weekends had lower risks of anxiety [63].

A total of 3 studies examined the associations between step count and anxiety. One study found that lower daily step count and lower SD of daily step count were associated with more anxiety symptoms [58]. A longitudinal study found that higher step count was associated with lower anxiety symptoms [60]; another longitudinal study found no association [64].

Of the 5 studies investigating other activity metrics (average daily exercise duration, total sedentary time on weekdays and weekends, and overall physical activity) [37,44,47,58,66], only 2 did not find associations with anxiety symptoms [44,47]. More physical activity was associated with lower odds of current and lifetime GAD [37] and lower anxiety symptoms [37,66].

#### Heart Rate

A total of 5 studies investigated the association between heart rate metrics and anxiety symptoms [58,61,64-66]. Four studies investigated the association between HRV metrics and anxiety [61,64-66]. HRV metrics can be examined in either the time-domain (measuring the amount of variability in intervals between heartbeats over time) or the frequency domain (measuring how HRV is distributed across different frequency bands). Within the frequency domain, the high-frequency component has been linked with parasympathetic nervous system (PNS) activation, and the low-frequency component has been linked with sympathetic nervous system (SNS) activation. Both central tendency and variability of metrics can be assessed in the HRV time and frequency domains.

One study focusing on the frequency domain found that, compared to a low trait anxiety group, the high trait anxiety group showed greater variability in HRV frequency metrics associated with PNS activation and the consistency of this activation, SNS activation, and the balance of PNS and SNS activation (eg, low-frequency/high-frequency HRV) in addition to daily changes of this balance [65]. Additionally, a higher mean and greater variance in daily changes in the balance of PNS and SNS activation were associated with increased odds of having trait anxiety [65]. In contrast, a separate study observed that higher mean low frequency (SNS activation) was associated with lower anxiety scores, and that mean high-frequency HRV (PNS activation) and the low frequency/high frequency ratio were not associated with anxiety symptoms [61]. This study also examined the association between HRV time-domain metrics and anxiety at 2 different time points, and found that higher mean deviation from the average HRV and short-term variations in heart rate were associated with lower anxiety scores [61]. One study found no association between HRV root mean square of successive differences and anxiety, a time-domain HRV metric that measures beat-to-beat changes in heart rate [64]. A study examined HRV but did not specify whether it was calculated in the time or frequency domain and found that higher HRV was associated with lower anxiety symptoms [66].

A total of 2 studies examined associations between average heart rate and anxiety, all reporting consistent findings. One study found that higher average heart rate and lower SD of heart rate were associated with more anxiety symptoms [58]. Another study found that individuals with anxiety had elevated heart rate compared with those without [64]. One study examined resting heart rate and found that higher levels were associated with more anxiety symptoms [66].

#### Other Metrics

One study found that individuals who were anxious had an elevated skin conductance response and skin temperature compared with those without anxiety [64].

### Qualitative Synthesis of Machine Learning Studies

Of the 8 studies included in this review, some studies used multiple algorithms within the same study, each with multiple performance metrics. Accuracy measures correct classifications (ie, presence/absence of anxiety). Sensitivity is the ability to detect true positives (ie, correct identification of individuals with anxiety), while precision is the correct classification among predicted positives (ie, how many individuals classified as having anxiety actually have anxiety). *F*_1_-score balances precision and sensitivity, and mean absolute error (MAE) measures the average prediction error, with lower MAE signifying better performance.

A total of 3 studies reported 13 accuracy estimates, ranging from 56.3% to 94.6% [70,71,73]. Two studies reported 9 sensitivity estimates (range 56.3%-94.6%), 9 precision estimates (range 56.3%-94.8%), and 9 *F*_1_-score estimates (range 55.9%-94.6%) [71,73]. Overall, 56 MAE estimates were reported from 2 studies [75,76], ranging from 0.05 to 0.63.

One article using 2 cohorts found better accuracy in predicting anxiety using frequency-domain HRV and nighttime HRV (range 63.9%-69.4%) data than time-domain HRV and daytime (range 63.2%-63.4%) data [70]. They also found that nighttime HRV data (maximum accuracy: 69.4%) performed better than daytime data (maximum accuracy: 63.2%) [70].

One study with older adults who had mild cognitive impairment found that combining activity (24-hour activity rhythm), sleep, and anxiety questionnaire items improved model performance across accuracy (91.0%-94.6% vs 56.3%-59.9%), precision (91.4%-94.8% vs 56.4%-61.0%), recall (91.0%-94.6% vs 56.3%-59.9%), and *F*_1_-scores (90.9%-94.6% vs 55.9%-59.0%) compared to models that excluded anxiety questionnaire data [73]. Similarly, another study using the same sample found that combining activity (step count) and sleep stages achieved a predictive success rate for participants without anxiety (98%) but had a low predictive success rate identifying those with anxiety (18%) [74]. Models including anxiety questionnaire items improved the predictive success rate for identifying participants with anxiety (82%) and maintained a good predictive success rate at identifying those without anxiety (95%). These studies did not report feature importance in their models.

A study with university students used digital biomarkers of health—HRV, electrodermal activity, and skin temperature—collected from wrist-worn wearables and smart shirts to predict low versus high anxiety levels. The best classifier (support vector machine) achieved an overall accuracy of 90.9%, with a precision of 0.90-0.92, recall of 0.90-0.92, and *F*_1_-score of 0.91 [72].

Two studies predicted anxiety in office workers [71,75]. One study, using 13 sleep metrics, achieved an *F*_1_-score of 75.6% and identified that the top 5 important predictors were REM sleep time ratio, REM sleep minutes, light sleep time ratio, deep sleep time ratio, and total wake time ratio [71]. Another study, using data from wearable devices (activity, stress, sleep, and heart rate data) and smartphones, found that the best non–time-based model (ie, without incorporating temporal data) predicted anxiety with an MAE of 0.6316. Time-based models showed improved performance, with an MAE of 0.3729 when using data from the previous 3 days to predict the next day’s anxiety [75]. They found that the top 20 predictors included measures of sleep (bedtime, wake time, and sleep duration), stress (low stress duration), and activity (highly active duration).

Finally, 1 study comparing 4 modeling approaches—conventional, multitask learning approach (model trained to solve multiple tasks simultaneously), time-based approach, and time-based multitask approach—found that time-based models using data from the previous 15 days to predict next day’s anxiety achieved the lowest MAE (0.0047), followed by the time-based multitask model (MAE 0.0083), the conventional model (MAE 0.1277), and the multitask model (MAE 0.1347) [76]. While activity, sleep, and heart rate were considered, the top 20 predictors did not include any of these metrics.

### Quantitative Synthesis of Cross-Sectional Studies

Random-effects meta-analyses were performed for studies that provided combinable effect sizes. All studies were cross-sectional and assessed associations between sleep metrics and anxiety symptoms.

**Figure 2:**
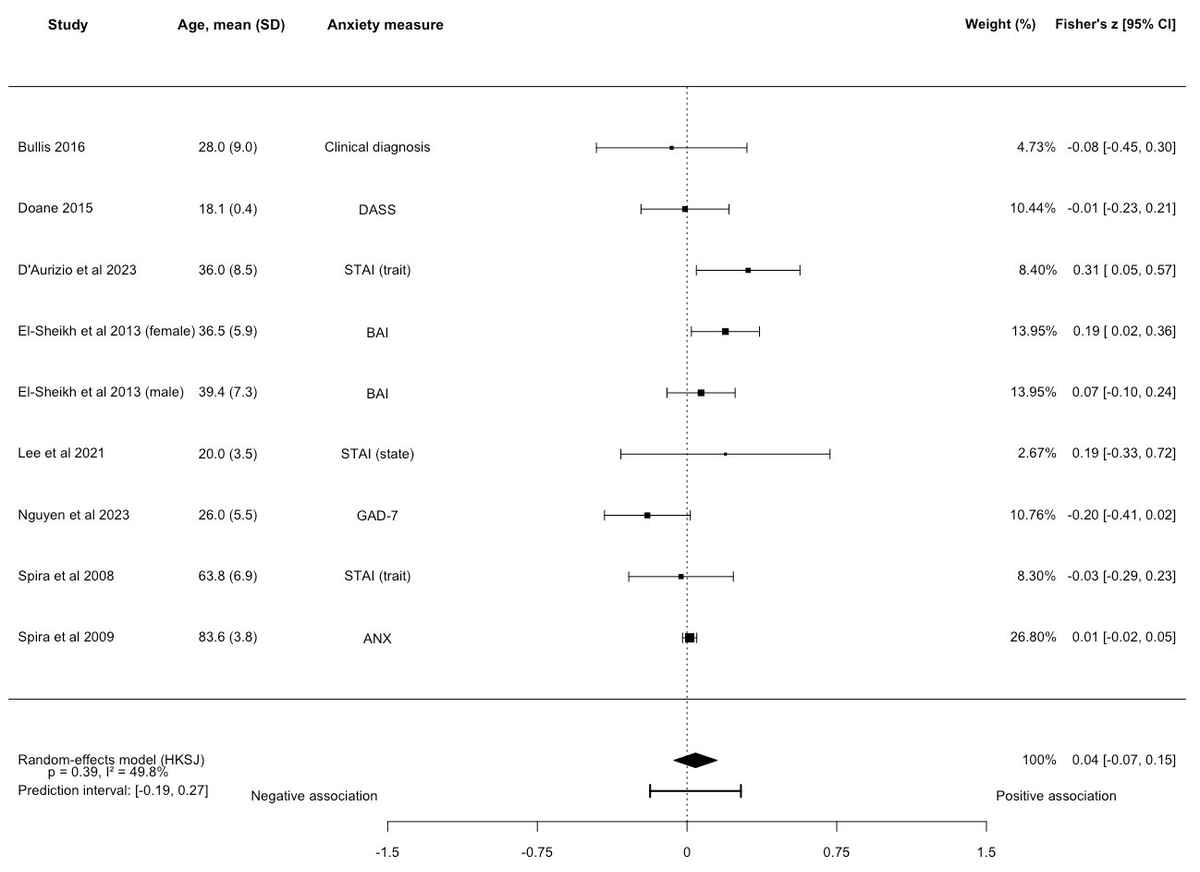
Forest plot of the associations between sleep onset latency and anxiety symptoms. Effect sizes are Fisher z with corresponding 95% CIs. No association between sleep onset latency and anxiety symptoms was observed (k=9, N=3643; Fisher z=0.04, 95% CI –0.07 to 0.15; *P*=.08). ANX: Goldberg Anxiety Scale; BAI: Beck Anxiety Inventory; DASS: Depression, Anxiety, and Stress Scale-Anxiety Subscale; GAD-7: Generalized Anxiety Disorder-7; HKSJ: Hartung-Knapp-Sidik-Jonkman; STAI: State-Trait Anxiety Inventory [36,39,41,42,48,50,51,52].

No association between SOL and anxiety symptoms was observed (k=9, N=3643; Fisher *z*=0.04, 95% CI –0.07 to 0.15; *P*=.08; Figure 2 [36,39,41,42,48,50-52] and Multimedia Appendix 6), with evidence of moderate to substantial heterogeneity between studies (*I^2^*=49.77%). The prediction interval ranged from –0.19 to 0.27. Around half of the included studies were rated as fair quality (k=5, 55.6%). The results remained unchanged in sensitivity analyses based on anxiety type (trait and state) and young adults (Multimedia Appendix 6). One study was not eligible for meta-analysis but found that higher SOL was associated with more anxiety symptoms [45].

**Figure 3:**
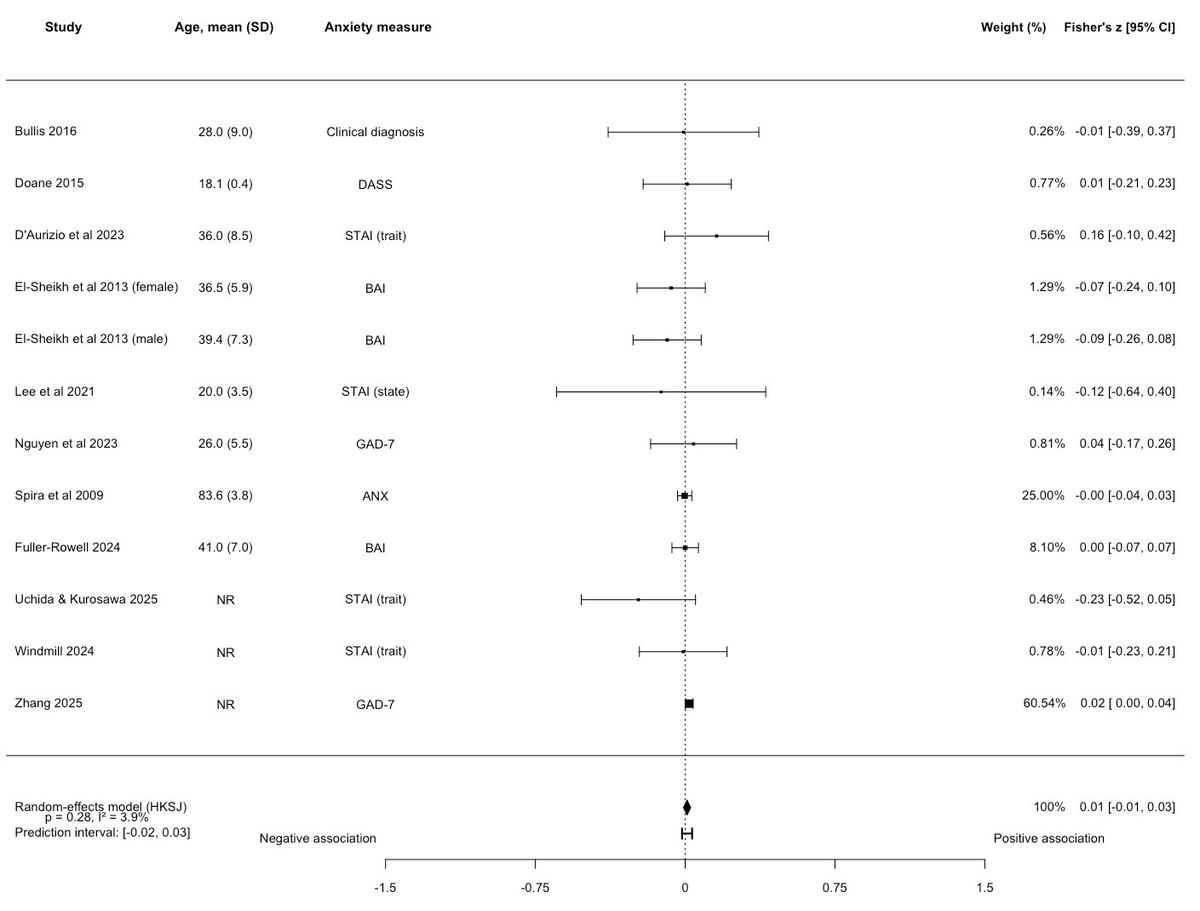
Forest plot of the association between total sleep time and anxiety symptoms. Effect sizes are Fisher z with corresponding 95% CIs. No association between total sleep time and anxiety symptoms was observed (k=12, N=14,721; Fisher z=0.009, 95% CI –0.01 to 0.03; *P*=.28). ANX: Goldberg Anxiety Scale; BAI: Beck Anxiety Inventory; DASS: Depression, Anxiety, and Stress Scale-Anxiety Subscale; GAD-7: Generalized Anxiety Disorder-7; HKSJ: Hartung-Knapp-Sidik-Jonkman; STAI: State-Trait Anxiety Inventory [36,39,41,42,45,48,50,52,55,56,58].

No association between TST and anxiety symptoms was observed (k=12, N=14,721; Fisher *z*=0.009, 95% CI –0.01 to 0.03; *P*=.28; Figure 3 [36,39,41,42,45,48,50,52,55,56,58] and Multimedia Appendix 6), with very low heterogeneity between studies (*I^2^*=3.89%). The prediction interval ranged from –0.02 to 0.03. The majority of the studies were rated as fair quality (k=9, 75%). The results remained unchanged in sensitivity analyses on anxiety type and young adults (Multimedia Appendix 6). Five studies were not eligible for quantitative synthesis. Findings from 4 studies aligned with those from the meta-analysis [38,46,53,66]. One study found a positive association between poor sleep—as measured by a composite including TST and other sleep metrics—and anxiety symptoms [43]. One study reported no association [49].

**Figure 4:**
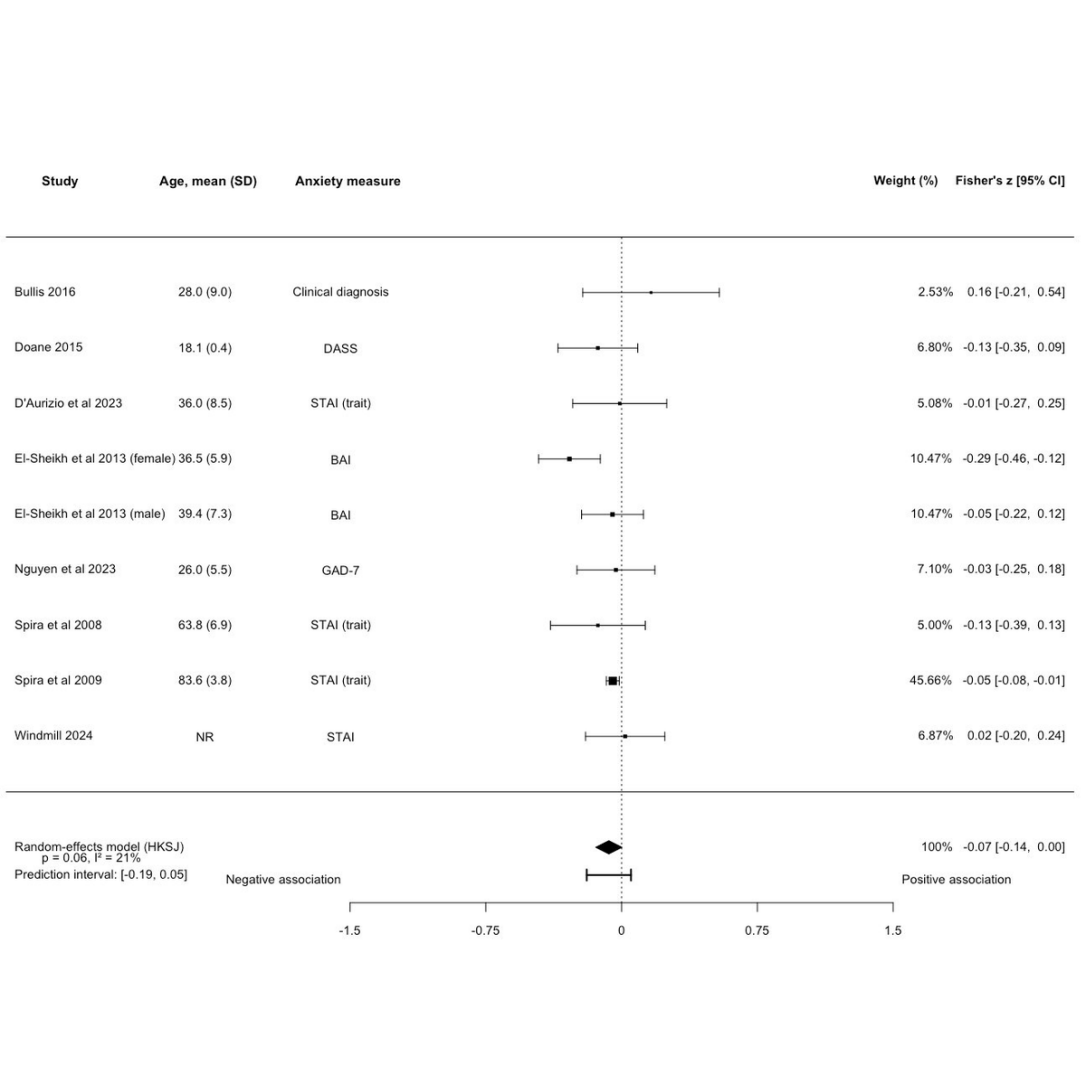
Forest plot of the association between sleep efficiency and anxiety symptoms. Effect sizes are Fisher z with corresponding 95% CIs. No association between sleep efficiency and anxiety symptoms (k=9, N=3710; Fisher z=-0.07, 95% CI –0.14 to 0.002; *P*=.06). BAI: Beck Anxiety Inventory; DASS: Depression, Anxiety, and Stress Scale-Anxiety Subscale; GAD-7: Generalized Anxiety Disorder-7; HKSJ: Hartung-Knapp-Sidik-Jonkman; STAI: State-Trait Anxiety Inventory [36,39,41,42,50,51,52,56].

No association between SE and anxiety symptoms was observed (k=9, N=3710; Fisher *z*=–0.07, 95% CI –0.14 to 0.002; *P*=.06; Figure 4 [36,39,41,42,50-52,56] and Multimedia Appendix 6), with low heterogeneity between studies (*I^2^*=20.97%). The prediction interval ranged from –0.19 to 0.05. The majority of studies were rated as fair quality (k=7, 77.8%). These results remained unchanged in sensitivity analyses on anxiety type and young adults (Multimedia Appendix 6). Of the 5 studies ineligible for quantitative synthesis, 3 found no association between SE and anxiety symptoms [45,49,57], 1 found a positive association [66], while 2 studies reported negative associations [43,77].

**Figure 5:**
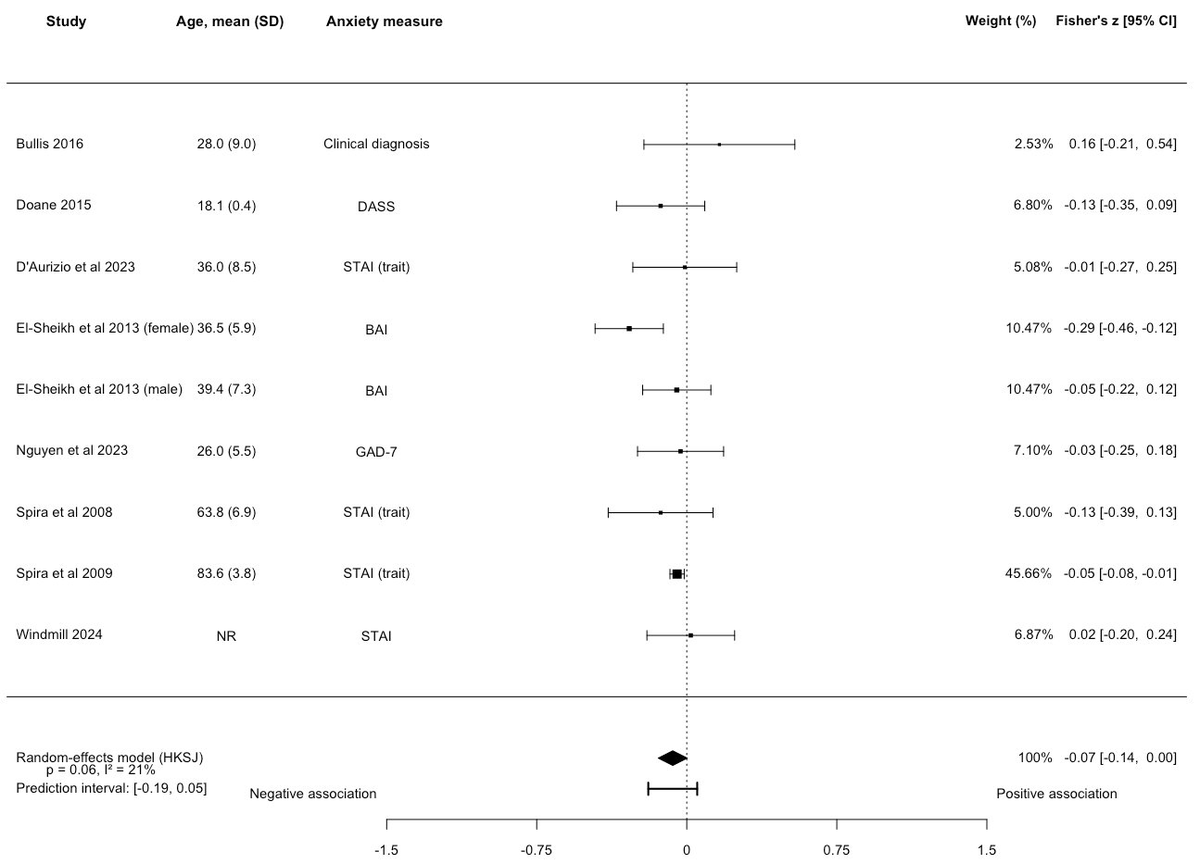
Forest plot of the association between wake after sleep onset and anxiety symptoms. Effect sizes are Fisher z with corresponding 95% CIs. No association between wake after sleep onset and more anxiety symptoms (k=6, N=3291; Fisher z=0.13, 95% CI –0.04 to 0.30; *P*=.11). ANX: Goldberg Anxiety Scale; GAD-7: Generalized Anxiety Disorder-7; HKSJ: Hartung-Knapp-Sidik-Jonkman; STAI: State-Trait Anxiety Inventory [36,39,48,50,51,52].

No association between WASO and anxiety symptoms was observed (k=6, N=3291; Fisher *z*=0.13, 95% CI –0.04 to 0.30; *P*=.11; Figure 5 [36,39,48,50-52] and Multimedia Appendix 6), with low to moderate heterogeneity between studies (*I^2^*=40.62%). The prediction interval ranged from –0.15 to 0.41. Most studies were rated as fair quality (k=4, 66.7%). These results remained unchanged in sensitivity analyses on anxiety type and young adults (Multimedia Appendix 6). One study that was not eligible for quantitative synthesis also found no association [49].

The Hartung-Knapp-Sidik-Jonkman–adjusted CIs reflect uncertainty around the average associations across studies, whereas the prediction intervals estimate the range of effects that may be observed in future studies conducted in different populations or settings. Across the sleep metrics examined, prediction intervals were generally similar to, but slightly wider than, the CIs, suggesting that effects observed in future studies are likely to be close to the average pooled estimates.

## Discussion

### Overview

This review aimed to advance scientific understanding of the association between digital biomarkers of health collected from wrist-worn devices and anxiety. Specific interest centered on determining which digital biomarkers of health have been the focus of existing research, and which demonstrate the strongest evidence of an association with anxiety. A total of 36 studies were included in this systematic review. Meta-analyses were conducted for 4 sleep metrics (SE, WASO, TST, and SOL), and none of these metrics were associated with anxiety. It was not possible to conduct meta-analyses for physical activity and heart rate metrics; however, qualitative synthesis suggests that lower physical activity levels and elevated heart rate were associated with higher levels of anxiety.

### Sleep

Our meta-analytic findings showed that SE, WASO, TST, and SOL were not statistically associated with anxiety. However, the association for SE was close to the significance threshold, suggesting a possible relationship. Heterogeneity was low for TST and SE, indicating that studies included in these meta-analyses found relatively consistent findings. In contrast, heterogeneity was moderate for WASO and SOL, suggesting that results varied across studies, potentially due to differences in populations, devices used to measure sleep metrics, or anxiety measurement. Subgroup analyses were not conducted as this was not prespecified in the protocol, and the number of studies contributing to each meta-analysis was small [78]. Finally, these findings should also be interpreted in the context of study quality. Most studies were rated as fair quality, reflecting some methodological limitations such as the lack of sample size calculation and no adjustment for key potential confounding variables.

The Pittsburgh Sleep Quality Index, commonly used to assess subjective sleep, comprises 7 components (sleep quality, sleep latency, sleep duration, SE, sleep disturbance, use of sleep medication, and daytime dysfunction) [79]. Existing research using the Pittsburgh Sleep Quality Index generally reports that shorter sleep duration, poor sleep quality, lower SE, and longer SOL are associated with anxiety disorders in both young and older adults [80-83]. In addition, worse subjective sleep quality has also been associated with more nonclinical levels of anxiety symptoms [84,85].

Research examining associations between anxiety and sleep metrics derived from polysomnography, the gold standard measurement of objective sleep, has been limited. One review identified 6 studies investigating polysomnographic sleep metrics, and found decreased SE and TST, and increased WASO in individuals with GAD compared with healthy controls [86]. Another review examined sleep metrics derived from electroencephalography and found that individuals with GAD had decreased TST, and increased WASO and SOL compared with healthy controls; however, no differences in SE were observed [16].

The lack of statistically significant associations observed in our meta-analyses contrasts with some findings from existing literature and may reflect differences in devices and methodological approaches. For instance, only 2 studies included in our meta-analysis asked participants to press an event marker to indicate when they began to fall asleep [36,87]. Without these event markers, estimating SOL may be less accurate [88], as different sleep behaviors can influence wrist movements. The majority of the studies (12/13, 92.3%) in our meta-analyses assessed anxiety symptoms, whereas the studies using polysomnography/electroencephalography focused on individuals with clinical anxiety. The lack of associations in our meta-analyses could also be attributed to differences in sample characteristics. Associations between sleep metrics and anxiety may emerge in clinical anxiety, but not in subthreshold anxiety symptoms.

### Physical Activity

Our review suggests that higher levels of physical activity are associated with lower anxiety symptoms. These findings appear to be largely consistent across different aspects of physical activity, including step count, total physical activity, and physical activity intensities. These findings align with existing reviews showing the protective effects of physical activity on reducing anxiety symptoms [89,90].

Our review found that sedentary behavior was associated with greater anxiety symptoms. The observed positive association between sedentary behavior and anxiety symptoms aligns with existing findings [91,92]. A meta-analysis primarily based on studies that used self-report questionnaires found a small positive association between sedentary behavior and anxiety [92]. Similar to our review, their study populations were largely composed of healthy individuals rather than those with anxiety disorders. It may be that stronger associations emerge with more severe anxiety. For example, 1 study included in our review observed a significant association only when assessing clinical vs nonclinical anxiety symptoms [46]. No association was observed when analyzing anxiety as a continuous variable (with a mean anxiety score below the clinical threshold) [46]. This potential threshold effect is further supported by another study included in our review, which found that individuals with more severe anxiety symptoms engaged in more sedentary behavior than those with no significant anxiety symptoms [40].

Regarding physical intensities, the majority of studies examining light physical activity (*k*=3, 75%) and MVPA (*k*=3, 75%) found that spending more time in these intensities was associated with lower anxiety symptoms. Similarly, a meta-analysis of physical activity interventions found that these interventions reduced anxiety symptoms [93]. Although the number of studies within each subgroup is limited, subgroup analyses of exercise intensity found that both moderate- and high-intensity exercise reduced anxiety symptoms [93]. Taken together, our review’s findings on physical activity intensities align with existing research showing that higher-intensity physical activity is effective in reducing anxiety symptoms.

### HRV

Our review found inconclusive evidence on the association between HRV and anxiety, as only 4 studies included in this review examined HRV. An existing meta-analysis on HRV found that individuals with GAD had reduced high-frequency and time-domain HRV [94], which means they may have less flexibility in adapting to stress. This partially aligns with the findings from 2 studies included in this review: 1 found that higher HRV (domain not specified) was associated with anxiety symptoms [66], while another found that higher mean time-domain HRV was associated with lower anxiety symptoms [61]. This study also found that greater high-frequency HRV variability was associated with more anxiety symptoms [61]. The discrepancy in findings may be due to the focus of the existing meta-analysis on clinical anxiety, whereas the 2 studies in our review examined anxiety symptoms.

Elevated resting or average heart rate reflects chronic SNS activation (SNS regulates the body’s fight-or-flight response) [95]. Compared to HRV, far fewer studies have examined average heart rate or resting heart rate in relation to anxiety. Existing evidence suggests that a higher resting heart rate is linked to greater anxiety symptoms and anxiety disorders [96,97]. Consistent with this literature, our review found that higher average heart rate and resting heart rate were associated with more anxiety symptoms.

### Studies Using Machine Learning Approaches

Machine learning approaches emphasize building models that generalize to new data. These approaches offer advantages over inferential statistical methods [98]. For example, machine learning approaches can capture both linear and nonlinear relationships between variables without strong assumptions about variable relationships. However, machine learning models can be challenging to interpret due to their “black box” nature, where decision-making processes are not transparent [99].

In this review, machine learning models predicting anxiety had accuracies ranging from 56.3% to 90.9%. The accuracy reported here is somewhat comparable to the 2 reviews on machine learning models using any wearable devices to predict depression (70%-89%) [17] and anxiety (82%) [18], which used a range of predictors (eg, heart rate, activity, sleep, audio, and skin temperature). The majority of studies included in this review did not include demographic predictors, which might further enhance model accuracy. Indeed, some studies in the depression and anxiety machine learning reviews used demographic data as predictors, further supporting the potential use of these predictors to improve the prediction of anxiety. The model with the highest performance (accuracy of 90.9%) combined digital biomarkers of health from more than 1 type of digital device (wrist-worn wearable and smart shirts) [72]. This aligns with existing research showing that models integrating digital biomarkers of health from multiple devices achieved better predictive performance than those relying on a single device [20]. For studies using only digital biomarkers of health from wrist-worn wearables in this review, the models with the highest accuracy included anxiety questionnaires as a predictor to predict clinically diagnosed anxiety. This likely inflates model performance due to circularity. When excluding models that incorporated anxiety questionnaires as inputs, the studies using only digital biomarkers of health from wrist-worn wearables reported accuracies ranging from 56.3% to 69.4%.

### Wrist-Worn Wearable Devices

Digital biomarkers from wrist-worn wearables offer several benefits over traditional assessments of anxiety, as they allow for continuous data collection over long periods without the need for repeated administration. In addition, these largely affordable and minimally intrusive devices can be used in natural settings, providing valuable insights into sleep and activity habits. While both research-grade and consumer-grade devices are less precise than polysomnography in measuring sleep [100-102], this may be due to their indirect measurement (eg, based on movement). Despite this limitation, they are generally considered valid tools for sleep assessment [100,103]. Moreover, polysomnography is neither cost-effective nor practical for large-scale real-world studies. Given that the majority of the studies in our review used research-grade devices, a key remaining question is whether these findings generalize to consumer-grade wrist-worn devices, which are more widely accessible to the general population.

There are also benefits to using self-report data. Self-report questionnaires can capture subjective experiences, such as perceived levels of exercise or sleep quality, which wearables cannot measure. Further, they have the potential to record underlying reasons for poor sleep, for example, bad dreams or pain. Therefore, while wrist-worn wearables offer advantages, incorporating self-report measures can provide a more comprehensive understanding of both objective and subjective experience.

### Strengths, Limitations, and Future Directions

To the best of our knowledge, this is the first review to examine the association between digital biomarkers of health from wrist-worn wearables and anxiety. We used double screening for all studies to ensure a rigorous selection process and to minimize bias. We also included gray literature to reduce potential publication bias and provide a more comprehensive review of existing studies.

This review also has several limitations. First, most studies focused on young adults. Part of the impetus for this review was to explore whether metrics from wrist-worn wearables could eventually be used to screen for dementia risk, as indicated by changes in anxiety symptoms. This is because anxiety is a potentially modifiable factor associated with dementia risk. However, the number of studies conducted in older adults is scarce. Future studies involving older adults would enrich our understanding of the association between digital biomarkers of health and anxiety in the context of dementia. Second, despite previous literature showing associations between physical activity and anxiety [104,105], few studies in our review focused on activity-related metrics. This precluded a meta-analytic synthesis of these findings. Wrist-worn devices generally provide more objective measurements of activity compared to self-report, which can be influenced by different biases [106], highlighting the need for further research. Third, although our search strategy and eligibility criteria allowed inclusion of any metrics measured by wrist-worn wearables, the majority of studies focused on sleep metrics. The search strategy was not peer-reviewed, and although it is possible that consulting with an information scientist might have led to the inclusion of additional relevant studies, this gap highlights an opportunity for future research to explore a wider range of metrics that could be associated with anxiety. Fourth, while machine learning has potential in predicting anxiety, the quality of these studies needs improvement. For example, future studies need to carefully consider the predictors used. None of the included machine learning studies considered demographics as potential predictors, despite existing research indicating sex differences in anxiety [107]. In addition, future studies should increase the interpretability of machine learning models by routinely reporting Shapley additive explanations values or feature importance scores. Additionally, most of the studies focused on a single metric (eg, sleep), despite the potential for the models to include multiple predictors. More research using machine learning approaches is needed, especially to compare whether this approach is superior to traditional approaches that use inferential statistics in predicting anxiety.

Wrist-worn wearable devices offer a scalable and noninvasive method to passively and continuously monitor anxiety symptoms. Metrics collected from these devices have the potential to serve as a screening or detection tool for anxiety, allowing individuals at risk of developing clinical anxiety to be identified before consulting a clinician. If these metrics and models have high predictive accuracy, they could support clinicians in identifying at-risk individuals and provide timely assessments and interventions. Before such an approach can be implemented in real-world clinical practice, more research is essential. In order to develop a clinically meaningful prediction model, interdisciplinary collaboration is crucial (eg, clinicians or machine learning experts). This will ensure the models are accurate, interpretable, clinically meaningful, and integrated into the health care system rather than remaining a research-focused tool.

### Conclusions

This review is the first to comprehensively synthesize evidence on the association between digital biomarkers of health derived from wrist-worn wearable devices and anxiety, integrating findings from both inferential statistical and machine learning studies. By systematically synthesizing the literature, this review identified gaps in evidence for future research.

Our meta-analyses found no associations between SOL, TST, SE, and WASO and anxiety. Although the number of studies was limited, there was evidence that lower physical activity levels and elevated heart rate were associated with more anxiety symptoms. Existing studies on light physical activity, MVPA, and HRV present inconsistent or inconclusive findings, highlighting the need for more research. Further, a comprehensive review of the literature yielded few machine learning studies in this area, and their quality was variable. While machine learning is increasingly used to analyze wearable data, its application for predicting anxiety remains in the early stages.

Machine learning and digital biomarkers could become powerful tools for early detection of anxiety, symptom monitoring and management, and prevention, particularly when used as part of broader assessment frameworks. Continuous data collection could support long-term monitoring of behavioral and physiological patterns relevant to anxiety, and may complement self-report and clinical assessments rather than being used as stand-alone screening tools. Given that anxiety is a potentially modifiable psychological factor associated with dementia risk, wearable-derived digital biomarkers may also offer future opportunities to support early identification and monitoring of dementia risk. However, further high-quality research is required before such approaches can be meaningfully translated into clinical or public health practice.

## Appendix

Multimedia Appendix 1 PRISMA 2020 Checklist.

Multimedia Appendix 2 PRISMA 2020 for abstracts checklist.

Multimedia Appendix 3 PRISMA-S Checklist.

Multimedia Appendix 4 Overview of search terms.

Multimedia Appendix 5 Risk of bias studies.

Multimedia Appendix 6 Meta-analytic results for sleep metrics.

## Abbreviations

GAD: generalized anxiety disorder
HRV: heart rate variability
MAE: mean absolute error
MVPA: moderate to vigorous physical activity
PECOS: Population, Exposure, Control, Outcome, and Study design
PNS: parasympathetic nervous system
PRISMA: Preferred Reporting Items for Systematic Reviews and Meta-Analyses
PRISMA-S: Preferred Reporting Items for Systematic Reviews and Meta-Analyses–Search Extension
PROSPERO: International Prospective Register of Systematic Reviews
REM: rapid eye movement
SE: sleep efficiency
SNS: sympathetic nervous system
SOL: sleep onset latency
TST: total sleep time
WASO: wake after sleep onset

## Data Availability

The dataset generated and analyzed during this study is available from the corresponding author on reasonable request.
