# Supplementary material for: Association Between Digital Biomarkers of Health and Anxiety: Systematic Review and Meta-Analysis
Source: J Med Internet Res. 2026 Mar 9;28:e73812. doi: 10.2196/73812 (PMC13010082; doi:10.2196/73812)
Supplement: Multimedia Appendix 4 [file jmir_v28i1e73812_app4.docx]

**Overview of search terms**

**Medline**

|  | **Search terms** |
| --- | --- |
| **Digital biomarkers** | (wearable* or "digital biomarker*" or smartwatch* or "smart watch" or "apple watch" or applewatch or fitbit or Samsung or garmin or lg or Huawei or sony or xiaomi or adidas or nike or axivity or pedometer* or "fitness track*" or "fitness monitor*" or acceleromet* or "activity monitor*" or "activity track*" or actigraphy).ti,ab,kw |
| **Psychosocial dementia risk factors** | (depress* or anxiet* or anxious* or "post-traumatic stress disorder" or PTSD or lonel* or "social isolation" or psychosis or psychotic or schizophreni* or bipolar).ti,ab,kw |

**Embase**

|  | **Search terms** |
| --- | --- |
| **Digital biomarkers** | (wearable* or "digital biomarker*" or smartwatch* or "smart watch" or "apple watch" or applewatch or fitbit or Samsung or garmin or lg or Huawei or sony or xiaomi or adidas or nike or axivity or pedometer* or "fitness track*" or "fitness monitor*" or acceleromet* or "activity monitor*" or "activity track*" or actigraphy).ti,ab,kw |
| **Psychosocial dementia risk factors** | (depress* or anxiet* or anxious* or "post-traumatic stress disorder" or PTSD or lonel* or "social isolation" or psychosis or psychotic or schizophreni* or bipolar).ti,ab,kw |

**PsycINFO**

|  | **Search terms** |
| --- | --- |
| **Digital biomarkers** | (wearable* or "digital biomarker*" or smartwatch* or "smart watch" or "apple watch" or applewatch or fitbit or Samsung or garmin or lg or Huawei or sony or xiaomi or adidas or nike or axivity or pedometer* or "fitness track*" or "fitness monitor*" or acceleromet* or "activity monitor*" or "activity track*" or actigraphy).ti,ab,id |
| **Psychosocial dementia risk**  **factors** | (depress* or anxiet* or anxious* or "post-traumatic stress disorder" or PTSD or lonel* or "social isolation" or psychosis or psychotic or schizophreni* or bipolar).ti,ab,id |

**Web of Science**

|  | **Search terms** |
| --- | --- |
| **Digital biomarkers** | wearable*or "digital biomarker*" or smartwatch* or "smart watch" or "apple watch" or applewatch or fitbit or Samsung or garmin or lg or Huawei or sony or xiaomi or adidas or nike or axivity or pedometer* or "fitness track*" or "fitness monitor*" or acceleromet* or "activity monitor*" or "activity track*" or actigraphy |
| **Psychosocial dementia risk**  **factors** | depress* or anxiet* or anxious* or "post-traumatic stress disorder" or PTSD or lonel* or "social isolation" or psychosis or psychotic or schizophreni* or bipolar |

**CINAHL**

|  | **Search terms** |
| --- | --- |
| **Digital biomarkers** | TI wearable* or AB wearable* or TI "digital biomarker*" or AB "digital biomarker*" or TI smartwatch* or AB smartwatch* or TI "smart watch" or AB "smart watch" or TI "apple watch" or AB "apple watch" or TI applewatch or AB applewatch or TI fitbit or AB fitbit or TI Samsung or AB Samsung or TI garmin or AB garmin or TI lg or AB lg or TI Huawei or AB Huawei or TI sony or AB sony or TI xiaomi or AB xiaomi or TI adidas or AB adidas or TI nike or AB nike or TI axivity or AB axivity or TI pedometer* or AB pedometer* or TI "fitness track*" or AB "fitness track*" or TI "fitness monitor*" or AB fitness monitor*" or TI acceleromet* or AB acceleromet* or TI "activity monitor*" or AB "activity monitor*" or TI "activity track*" or AB "activity track*" or TI actigraphy or AB actigraphy |
| **Psychosocial dementia risk**  **factors** | TI depress* or AB depress* or TI anxiet* or AB anxiet*or TI anxious* or AB anxious* or TI "post-traumatic stress disorder" or AB "post-traumatic stress disorder" or TI PTSD or AB PTSD or TI lonel* or AB lonel* or TI "social isolation" or AB "social isolation" or TI psychosis or AB psychosis or TI psychotic or AB psychotic or TI schizophreni* or AB schizophreni* or TI bipolar AB bipolar |

**ProQuest Dissertations & Theses Global**

|  | **Search terms** |
| --- | --- |
| **Digital biomarkers** | (wearable* or "digital biomarker*" or smartwatch* or "smart watch" or "apple watch" or applewatch or fitbit or Samsung or garmin or lg or Huawei or sony or xiaomi or adidas or nike or axivity or pedometer* or "fitness track*" or "fitness monitor*" or acceleromet* or "activity monitor*" or "activity track*" or actigraphy) |
| **Psychosocial dementia risk factors** | (depress* or anxiet* or anxious* or "post-traumatic stress disorder" or PTSD or lonel* or "social isolation" or psychosis or psychotic or schizophreni* or bipolar) |
