# Supplementary material for: Association Between Digital Biomarkers of Health and Anxiety: Systematic Review and Meta-Analysis
Source: J Med Internet Res. 2026 Mar 9;28:e73812. doi: 10.2196/73812 (PMC13010082; doi:10.2196/73812)
Supplement: Multimedia Appendix 5 [file jmir_v28i1e73812_app5.docx]

**Risk of bias for studies using inferential statistical approaches**

| **Criteria** | **Yes** | **No** | **Other**  **(CD, NR, NA)** |
| --- | --- | --- | --- |
| 1. Was the research question or objective in this paper clearly stated? | 36 (100%) | 0 (0%) | 0 (0%) |
| 2. Was the study population clearly specified and defined? | 36 (100%) | 0 (0%) | 0 (0%) |
| 3. Was the participation rate of eligible persons at least 50%? | 26 (72.2%) | 2 (5.6%) | 8 (22.2%) |
| 4. Were all the subjects selected or recruited from the same or similar populations (including the same time period)? Were inclusion and exclusion criteria for being in the study prespecified and applied uniformly to all participants? | 36 (100%) | 0 (0%) | 0 (0%) |
| 5. Was a sample size justification, power description, or variance and effect estimates provided? | 0 (0%) | 36 (100%) | 0 (0%) |
| 6. For the analyses in this paper, were the exposure(s) of interest measured prior to the outcome(s) being measured? | 11 (30.6%) | 1 (2.8%) | 24 (66.7%) |
| 7. Was the timeframe sufficient so that one could reasonably expect to see an association between exposure and outcome if it existed? | 14 (38.9%) | 0 (0%) | 22 (61.1%) |
| 8. For exposures that can vary in amount or level, did the study examine different levels of the exposure as related to the outcome (e.g., categories of exposure, or exposure measured as continuous variable)? | 36 (100%) | 0 (0%) | 0 (0%) |
| 9. Were the exposure measures (independent variables) clearly defined, valid, reliable, and implemented consistently across all study participants? | 36 (100%) | 0 (0%) | 0 (0%) |
| 10. Was the exposure(s) assessed more than once over time? | 12 (33.3%) | 2 (5.6%) | 22 (61.1%) |
| 11. Were the outcome measures (dependent variables) clearly defined, valid, reliable, and implemented consistently across all study participants? | 36 (100%) | 0 (0%) | 0 (0%) |
| 12. Were the outcome assessors blinded to the exposure status of participants? | 1 (2.8%) | 0 (0%) | 35 (97.2%) |
| 13. Was loss to follow-up after baseline 20% or less? | 8 (22.2%) | 0 (0%) | 28 (77.8%) |
| 14. Were key potential confounding variables measured and adjusted statistically for their impact on the relationship between exposure(s) and outcome(s)? | 21 (58.3%) | 15 (41.7%) | 0 (0%) |

*Abbreviations:* CD, cannot determine; NA, not applicable; NR, not reported

*Notes:* Data presented as *k* (%). Criteria 6, 7, 10, and 13 were excluded for cross-sectional studies.

**Risk of bias studies using machine learning approaches**

|  | Risk of bias | | | | Applicability concerns | | |
| --- | --- | --- | --- | --- | --- | --- | --- |
|  | Participants | Index test | Reference standard | Analysis | Participants | Index test | Reference standard |
| Coutts et al., 2020 Trial 1 [70] | High risk | Low risk | Low risk | Low risk | Low concerns | Low concerns | Low concerns |
| Coutts et al., 2020 Trial 2 [70] | Low risk | Low risk | Low risk | Low risk | Low concerns | Low concerns | Low concerns |
| Fukuda et al., 2020 [71] | High risk | Unclear | Unclear | Unclear | Low concerns | Low concerns | Low concerns |
| He et al, 2025 [72] | High risk | Low risk | Low risk | Low risk | Low concerns | Low concerns | Low concerns |
| Lee et al., 2022 [73] | High risk | Low risk | Unclear | Low risk | Low concerns | Low concerns | Low concerns |
| Lee et al., 2024 [74] | High risk | Low risk | Unclear | Low risk | Low concerns | Low concerns | Low concerns |
| Saylam et al., 2023 [75] | Unclear | Low risk | Unclear | Low risk | Unclear | Low concerns | Low concerns |
| Saylam et al., 2024 [76] | Unclear | Low risk | Unclear | Unclear | Unclear | Low concerns | Low concerns |
