# Supplementary material for: Association Between Digital Biomarkers of Health and Anxiety: Systematic Review and Meta-Analysis
Source: J Med Internet Res. 2026 Mar 9;28:e73812. doi: 10.2196/73812 (PMC13010082; doi:10.2196/73812)
Supplement: Multimedia Appendix 6 [file jmir_v28i1e73812_app6.docx]

**Meta-analytic results for sleep metrics**

|  | *K* | N | ES (z) | 95% CI | p-value | I^2^ | Tau^2^ |
| --- | --- | --- | --- | --- | --- | --- | --- |
| **SOL** |  |  |  |  |  |  |  |
| Primary | 9 | 3643 | 0.04 | -0.07, 0.15 | .08 | 49.77% | 0.01 |
| Trait | 7 | 3544 | 0.04 | -0.10, 0.19 | .48 | 63.55% | 0.11 |
| State | 4 | 218 | 0.12 | -0.27, 0.51 | .38 | 67.75% | 0.04 |
| Young adults | 7 | 545 | 0.06 | -0.10, 0.23 | .38 | 54.19% | 0.02 |
| **TST** |  |  |  |  |  |  |  |
| Primary | 12 | 14712 | 0.009 | -0.01, 0.03 | .28 | 3.89% | 0.01 |
| Trait | 10 | 14622 | 0.01 | -0.01, 0.03 | .32 | 4.74% | 0.01 |
| State | 3 | 159 | 0.10 | -0.40, 0.61 | .47 | 46.54% | 0.02 |
| Young adults | 9 | 678 | -0.03 | -0.11, 0.04 | .33 | 0% | 0 |
| **SE** |  |  |  | å |  |  |  |
| Primary | 8 | 3710 | -0.07 | -0.14, 0.002 | .06 | 20.97% | 0.04 |
| Trait | 8 | 3628 | -0.07 | -0.15, 0.02 | .10 | 27.08% | 0.05 |
| State | 3 | 201 | -0.09 | -0.45, 0.28 | .42 | 21.36% | 0.004 |
| Young adults | 7 | 611 | -0.08 | -0.20, 0.05 | .17 | 34.29% | 0.08 |
| **WASO** |  |  |  |  |  |  |  |
| Primary | 6 | 3291 | 0.13 | -0.04, 0.30 | .30 | 40.62% | 0.01 |
| Trait | 5 | 3274 | 0.11 | -0.06, 0.28 | .15 | 35.43% | 0.01 |
| State | 3 | 136 | 0.24 | -0.34, 0.82 | .21 | 51.98% | 0.03 |
| Young adults | 4 | 193 | 0.13 | -0.26, 0.53 | .24 | 49.51% | 0.03 |

*Abbreviations:* *K*, number of studies; N, sample size, ES, Effect size; z, Fisher’s z; 95% CI, 95% confidence interval; Tau^2^, Between-study variance; I^2^, proportion of observed dispersion due to real variation in effect size
